# The Interaction between Obstructive Sleep Apnea and Parkinson's Disease: Possible Mechanisms and Implications for Cognitive Function

**DOI:** 10.1155/2015/849472

**Published:** 2015-10-05

**Authors:** Marta Kaminska, Anne-Louise Lafontaine, R. John Kimoff

**Affiliations:** ^1^Respiratory Division & Sleep Laboratory, McGill University Health Centre, Montreal, QC, Canada H4A 3J1; ^2^Respiratory Epidemiology and Clinical Research Unit, McGill University Health Centre, Montreal, QC, Canada H4A 3J1; ^3^Montreal Neurological Hospital, McGill University Health Centre, Montreal, QC, Canada H3A 2B4

## Abstract

Parkinson's disease (PD) is a relentlessly progressive neurodegenerative disorder associated with hallmark motor and nonmotor symptoms (NMS) such as sleep disturbances and cognitive dysfunction. While dopaminergic treatments have improved the motor aspects of PD, progression remains inevitable. Research has recently increasingly focused on strategies to modify disease progression and on nonmotor manifestations of PD, given their impact on patients' quality of life. Obstructive sleep apnea (OSA) is a treatable sleep disorder, common in the general population, associated with excessive daytime sleepiness and neurocognitive deficits. Neuroimaging has demonstrated structural and functional changes in OSA patients; in animal models, OSA causes brain inflammation and oxidative injury, including in key areas involved in PD pathophysiology such as locus coeruleus. The prevalence of OSA in PD has been variable in studies to date, and potential consequences and interrelationship between the two disorders have not been well studied. There is however emerging evidence that OSA is associated with increased NMS in PD, particularly cognitive dysfunction. This review focuses on the possible interrelationship between OSA and PD. Mechanisms promoting OSA in PD will be reviewed, as well as mechanisms whereby OSA can affect the neurodegenerative process in PD.

## 1. Introduction

**Figure 1:**
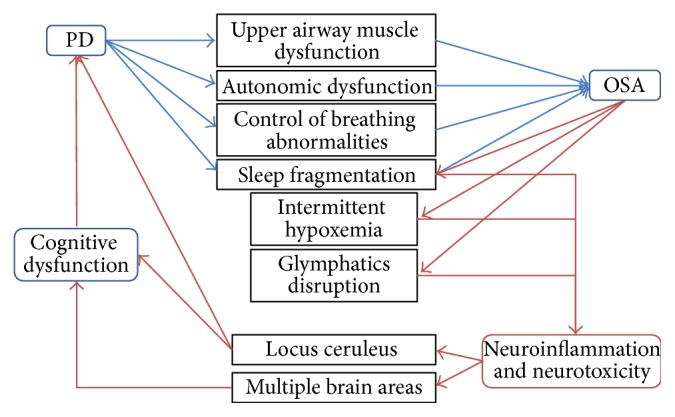
Hypothetical mechanistic relationship between PD and OSA. Legend. PD: Parkinson's disease; OSA: obstructive sleep apnea.

Parkinson's disease (PD) is the second most frequent neurodegenerative disorder, and its prevalence is expected to increase as the population ages [1]. Obstructive sleep apnea (OSA) is a treatable sleep disorder that is common in the general population and is associated with adverse outcomes including cognitive dysfunction [2]. OSA results in sleep fragmentation and intermittent hypoxemia that can have significant detrimental consequences on the brain. However, OSA prevalence in PD has been variable in studies to date in part due to methodological variability, such that until recently, OSA has not been perceived to be a significant issue in PD. Thus, to date, the potential consequences and interrelationship between OSA and PD have not been well studied. However, when already affected by a degenerative process like PD, one could speculate that the brain may be more vulnerable to the effects of OSA due to reduced ability to compensate and also more responsive to OSA treatment. In this paper, we explore the possible bidirectional relationship between OSA and PD (Figure 1). We review the possible pathophysiologic factors predisposing to OSA in the context of PD. We then review the known consequences of OSA on the brain. These data suggest that OSA may play a significant role in the neurodegenerative process of PD, particularly as it relates to cognitive dysfunction.

## 2. OSA Overview

OSA is characterized by recurrent complete (apnea) or partial (hypopnea) upper airway obstruction resulting in intermittent hypoxemia and arousals from sleep. Pathophysiologic factors include reduced airway dimensions, altered central control of breathing, sleep-wake instability, altered arousal responsiveness, and upper airway dilator muscle dysfunction. The latter maintain upper airway patency and are modulated by neuronal inputs related to sleep-wake state, mechanoreceptor input, blood gases, autonomic activity, and other factors [3]. The prevalence of OSA depends on the definition of respiratory events used [4, 5] and significantly increases with age. In the general population, OSA prevalence has been estimated at 9–47% of women and 17–52% of men aged 50–70 years [6, 7]. Indeed, hypopneas in the original Wisconsin cohort study were scored as such only in the presence of a drop in hemoglobin oxygen saturation [6]. Currently recommended criteria include hypopneas associated with arousal only [8], which would lead to higher prevalence estimates [9]. OSA has been associated with a range of adverse outcomes including cognitive impairment, increased risk of hypertension, diabetes, fatal and nonfatal coronary events, arrhythmias such as atrial fibrillation and nonsustained ventricular tachycardia, congestive heart failure, stroke, and mortality (reviewed in [10]).

## 3. OSA in Other Neurological Disorders

OSA risk is increased with male sex, older age, and higher body mass index (BMI), but also in conditions such as neuromuscular disorders [11], epilepsy [12], multiple sclerosis [13], and stroke [14]. Moreover, in CNS disorders, OSA appears to modify the manifestations or disease course, which suggests a bidirectional relationship. For example, OSA is associated with an increased incidence of stroke [15, 16]. In turn, OSA appears to be associated with poorer outcome at discharge and up to 12 months and increased mortality at 12 months after stroke [17]. Furthermore, despite the difficulty in applying continuous positive airway pressure (CPAP) therapy in patients soon after a stroke, functional outcomes were improved in patients treated for their OSA in two randomized controlled trials (RCT) [18, 19]. In MS, we reported that OSA was associated with increased fatigue [20], which is one of the most frequent, pervasive, and incapacitating symptoms of MS. Treatment of OSA led to improved fatigue [21]. OSA has also been associated with poor seizure control in epilepsy [12]. Patients with OSA who were compliant with CPAP had reduced seizure frequency [22].

## 4. OSA and Cognitive Function

In addition to sleepiness, OSA in the general population and in the elderly has been associated with impaired cognition and psychomotor performance [2, 23–26]. In women, this relationship is more pronounced among carriers of the APOE 4 genotype [26]. Most commonly reported deficits in OSA are reduced executive function and attention capacity deficits such as reduced information processing speed and short-term memory span, as well as deficient verbal fluency and impaired vigilance [27, 28]. Data from prospective studies have also demonstrated that individuals with OSA at baseline were more likely to develop cognitive impairment [29, 30] and frank dementia [31] at follow-up.

The response of neurocognitive dysfunction to CPAP therapy in the general population has been variable and incomplete [32–35]. This has been suggested to stem from near-normal cognitive function before CPAP, lack of statistical power [32, 35], poor compliance with treatment [36], or irreversible brain damage from long-standing OSA [37].

A recent meta-analysis evaluating the effect of CPAP on various subtypes of executive function found a significant beneficial effect [38]. However, the APPLES trial [35], a large multicenter RCT of CPAP versus sham CPAP which evaluated three domains of cognitive function in OSA (attention and psychomotor function, learning and memory, executive, and frontal lobe function), failed to show the expected benefits. In this study, individuals with a Mini-Mental State Examination score ≤ 26 (normal cutoff in healthy adults) were excluded. Hence, only those with scores within the normal range were included, and there was little room for further improvement. The authors advanced the “cognitive reserve theory” to explain lack of positive results. That is, some individuals may have greater preexisting flexibility in neural function and capacity to cope with disruption, or better compensatory mechanisms [39]. Possibly, then, a detrimental effect of OSA on cognition may only become apparent in individuals with reduced cognitive reserve, or with another predisposing condition to cognitive dysfunction.

In Alzheimer's disease (AD), data is relatively scant and inconclusive regarding a relationship with OSA. There is a suggestion that OSA is more prevalent in AD patients than in controls [40], and severity of dementia correlates with severity of OSA [41], but not all studies have found this and the magnitude of the effect does not appear to be very large [42]. Moreover, directionality of the relationship is unclear from these cross-sectional studies. A small trial of OSA treatment with CPAP in AD found that cognition improved with CPAP use in the treated group [43] and that there appeared to be slowed deterioration of cognition with sustained use of CPAP in the observational follow-up [44].

## 5. OSA Prevalence in PD and Possible Pathogenic Mechanisms

Sleep disturbances are frequent in PD and include insomnia, hypersomnia, sleep architecture and circadian abnormalities, restless legs syndrome, and REM Sleep Behavior Disorder (RBD) [45]. OSA is reported to occur in 20–60% of PD subjects [46–51]. This wide range likely reflects differences in patient populations, small sample sizes with selection bias, and most importantly differences in scoring of respiratory events between laboratories [5]. In particular, studies suggesting a low prevalence of OSA in PD have included only hypopneas with desaturations [48], overlooking entirely respiratory events causing sleep fragmentation without hypoxemia. It has also been suggested that OSA prevalence in more advanced PD might be reduced compared with the general population due to lower body mass index of PD patients [52]. However, this may depend on the criteria used to define OSA, as hypoxemia is more likely to be associated with a higher BMI. OSA in PD may not follow the same pattern as in the general population. Trotti and Bliwise did not find BMI to be correlated with OSA severity in PD [51]. Correlation between OSA severity and PD severity has been found in two studies [47, 50] and in our own work [53], though causality cannot be inferred from these cross-sectional studies. While OSA does not appear to be more common in PD than the general population, it is clear that the two conditions do not uncommonly coexist, either because OSA is frequent in the general population and thus coincides with PD, or due to PD-related changes predisposing to OSA, or both.

Biologic plausibility exists for PD itself being involved in OSA pathogenesis. The upper airway musculature may be affected by involuntary movements resulting in abnormal spirometry consistent with upper airway obstruction [54], which improves with levodopa [55]. These disturbances may be exacerbated in sleep, resulting in OSA. Our group has found that PD patients on night-time long-acting levodopa had less sleep-disordered breathing than those not on such medication [56]. This further supports the notion that the upper airway is responsive to levodopa and may thus be affected as part of the movement disorder, predisposing to OSA. Levodopa may also produce disordered breathing as a form of dyskinesia [57, 58].

PD is also associated with autonomic dysfunction, which may impair control of breathing, particularly during non-REM sleep where respiration is predominantly dependent on chemical drive. Such a mechanism has been suggested as a partial explanation of the high prevalence of sleep-disordered breathing in the Shy-Drager syndrome [59, 60]. Abnormal afferent chemosensitive feedback control to the central respiratory generator has been implicated [59]. This is consistent with reports of sleep-disordered breathing, occasionally fatal, occurring in patients undergoing cervical cordotomy for pain relief, which is associated with other manifestations of autonomic dysfunction [61]. OSA itself can alter autonomic function with consequences beyond the sleep period, particularly increased sympathetic tone that is associated with baroreflex and chemoreflex changes [62, 63]. Control of breathing is affected, potentially further promoting OSA. In PD, chemosensitivity to hypoxia was found to be reduced, despite normal pulmonary function, and this was associated with reduced dyspnea in hypoxic conditions [64]. Respiratory drive in response to hypercapnia was also found to be reduced [65], possibly as a result of involvement by the PD neurodegenerative process of the brainstem [66], where the central chemoreceptor and respiratory centers are located. An abnormal hypercapnic response can predispose to hypoventilation, especially in sleep. Moreover, activity of upper airway dilator muscles, a key element in OSA pathophysiology, is modulated by respiratory drive and CO_2_ levels [67, 68]. How these mechanisms affect the upper airway and respiration during sleep in patients with PD has not been directly studied.

Sleep fragmentation may itself induce respiratory disturbances. A change in sleep state such as the transition from wakefulness to sleep is associated with a change in respiration manifesting as periodic breathing, usually transient. However, in individuals with a low arousal threshold, a modest fluctuation in breathing may trigger an arousal. Arousals from sleep following a respiratory event lead to hyperpnea and hypocapnia, which in turn may trigger another respiratory pause upon return to sleep, triggering a cycle of respiratory instability, further promulgating OSA [69]. In mice, sleep fragmentation resulted in impaired arousal responses to hypercapnia [70], which could prolong apneas and hypopneas. In humans, sleep fragmentation led to increased upper airway collapsibility in sleep [71], increasing propensity for OSA. In PD, sleep fragmentation and dysfunction occur as part of the disease. This is thought to be multifactorial, due in part to dysfunctional sleep circuits but also to medications and comorbidities [72]. Hence the intrinsic sleep fragmentation in PD may be a factor in progression of OSA in this condition.

## 6. Mechanisms of Deleterious OSA Effects on the Brain

### 6.1. Intermittent Hypoxemia

The mechanisms involved in the effects of OSA on the brain in general and on cognitive function in particular have not been clearly elucidated, but several factors could play a role. OSA is increasingly being incriminated as causing neural injury. Intermittent* hypoxemia* in particular has been implicated, possibly through mechanisms of ischemia/reperfusion [73], and oxidative injury [74]. OSA with hypoxemia is also associated with delayed peripheral nerve conduction [75] and treatment of OSA partially reverses the dysfunction [76]. In animal models, exposure of rodents to intermittent hypoxemia resulted in impaired learning and memory that did not normalize after a recovery period. Increased astrocytes and neuronal apoptosis were found in frontal cortex areas (including cingulate gyrus) and certain hippocampal regions, implying differential neuronal susceptibility [77]. Reduction in striatal norepinephrine concentration was also shown as a result of intermittent hypoxemia [78], as well as injury in specific catecholaminergic neuron groups, notably the dopaminergic periaqueductal gray and locus coeruleus [79]. NADPH oxidase [80] and iNOS [73] were found to mediate this injury and the associated proinflammatory response. The proinflammatory transcription factor NF-*κ*B is also induced by intermittent hypoxemia in OSA [81, 82] causing systemic inflammation. Evidence of systemic inflammation in OSA was found with elevated plasma levels of C reactive protein [83], TNF-*α*, interleukin- (IL-) 6 [84, 85], and IL-8 [86]. IL-6 and TNF-*α* levels correlated with OSA severity [87]. This likely contributes to neuroinflammation [88] which promotes neurodegeneration [89, 90]. Although these OSA-related mechanisms might theoretically exacerbate PD neuropathology, they have not been studied to date in PD.

Intermittent hypoxemia in mice has also been found to be associated with reduced expression of brain-derived neurotrophic factor (BDNF) in the hippocampus and reduced long-term potentiation [91]. This could explain some cognitive deficits, as reduced BDNF levels have been associated with impaired cognition [92, 93]. However, in humans, serum BDNF levels were no different in OSA versus control subjects [94].

It should be noted that while OSA-related hypoxemia in humans has been associated with cognitive deficits in some studies [95, 96], others have found a paradoxical apparently protective effect [97]. Recent data suggest that there may be an ischemic preconditioning effect in some OSA patients [98]. Hence the exact role of hypoxemia as a cause of cognitive deficit in humans remains to be clarified, though severity of the intermittent hypoxia likely plays a role [88]. In PD hypoxemia associated with OSA is less marked as compared with non-PD individuals [48]. This is due to the lower BMI of PD patients with OSA. However, individuals earlier in the course of their PD may have a higher BMI, including before diagnosis, and hypoxemia might be a more important factor in that setting. Moreover, it is unknown what level of hypoxemia might be considered “safe” in PD. It is possible that what is inconsequential or protective in an otherwise healthy brain may be deleterious in PD. More research will be needed to clarify these relationships.

### 6.2. Sleep Fragmentation

In addition to hypoxemia, OSA is associated with* sleep fragmentation*, which appears to be a key factor in brain dysfunction and cognitive outcomes. Some deficits in OSA are similar to those occurring in sleep deprivation [99]. In a longitudinal study of elderly individuals, sleep fragmentation related to OSA, but not hypoxemia, was associated with cognitive decline [30]. Sleep fragmentation due to OSA was also found to be the best predictor of episodic memory deficits [100]. In mice, sleep fragmentation results in learning deficits. This was found to be associated with increased gene expression and activity of NADPH oxidase in the hippocampus and cortex of wild type mice [101]. However, mutant mice lacking NADPH oxidase activity were protected from the learning deficits. Chronic sleep fragmentation was also found to selectively increase cortical expression of TNF-*α* [102]. Moreover sleepiness and learning deficits associated with sleep fragmentation were absent in TNF-*α* double receptor knockout mice and in mice treated with a TNF-*α* neutralizing antibody [102]. Hence, sleep fragmentation appears to induce oxidative stress and inflammation just as intermittent hypoxia does. Interestingly, in a sleep fragmentation animal model of OSA, there was reduced neuronal excitability in the locus coeruleus [70], an area implicated in PD pathophysiology (compare with below).

### 6.3. Glymphatics

Recently a novel waste clearance system operating in the brain has been characterized, termed the glymphatic system [103]. It involves transport of CSF along periarterial spaces, via convective flow through the brain parenchyma and perivenous spaces into the cervical lymphatic system, eliminating soluble proteins and metabolites. Its function declines with age and this has been suggested to contribute to the accumulation of abnormal proteins in the extracellular space, such as *β*-amyloid or *α*-synuclein, rendering the brain more vulnerable to neurodegenerative pathologies. The particularity of this system is that it is activated only during sleep. Therefore, any process leading to sleep fragmentation can disrupt this system, resulting in potentially adverse consequences on brain homeostasis. It is known that dementia in PD often results from an “admixture of pathologies” [104–106], including Lewy body but also Alzheimer-related pathologies, with a smaller component of cerebrovascular pathology. One could therefore speculate that glymphatic abnormalities may be a nonspecific mechanism predisposing to cognitive dysfunction in PD. Glymphatics could be affected by sleep fragmentation or hemodynamic changes occurring in OSA. Intermittent hypoxia has also been implicated in potential blood-brain barrier dysfunction and alteration in brain water and solute fluxes, through a number of mechanisms stemming from a chronic maladaptive response [88].

### 6.4. Role of the Locus Coeruleus

The locus coeruleus has been implicated in cognitive decline in the general population. A recent autopsy study from a longitudinal clinical-pathologic cohort study on aging found that lower locus coeruleus neuronal density was associated with lower baseline level of cognition and faster cognitive decline [107]. An imaging study showed that locus coeruleus connectivity was correlated with memory scores and was reduced in patients with mild cognitive impairment [108]. With regards to PD, a recent case series and review by Del Tredici and Braak [104] focused on the role of noradrenergic defects in the locus coeruleus in development of dementia in PD.

The effects of intermittent hypoxemia and sleep fragmentation on the locus coeruleus and other specific brain regions, as described above, may have significant implications in PD. While the key abnormality in PD pathophysiology is loss of dopaminergic neurons of the substantia nigra, resulting in depletion of dopamine from the basal ganglia, other regions of neurodegeneration have been identified, which may better correlate with the nonmotor symptoms of PD [109, 110]. Locus coeruleus neurons specifically have been implicated in pathophysiology of PD: loss of their trophic influences may increase sensitivity of dopaminergic neurons to neurotoxic insults [111, 112]. The currently emerging concept of PD pathogenesis revolves around a combination of genetic, cellular, and environmental factors that independently or concomitantly result in cell death, possibly by triggering mitochondrial dysfunction and oxidative stress, abnormal protein degradation, and other forms of subcellular dysfunction [113]. After disease onset, regardless of the initial insult, the progression of cell loss may result from common pathways that include oxidative and nitrosative stress and neuroinflammation [113–115]. Neuroinflammation appears to play a key part in pathogenesis of PD. Nonsteroidal anti-inflammatory drugs decrease the risk of PD [116], and inflammatory cytokines are increased in the serum and/or cerebrospinal fluid of PD patients [IL-2, TNF*α*, IL-6, RANTES, osteopontin, and IL-1*β*]. In PD animal models, intranigral infusion of TNF*α* blockers attenuated dopaminergic neurodegeneration, while mice lacking TNF receptors 1 and 2 had attenuated striatal damage after injection of MPTP [115]. In the process of neuroinflammation, microglia became activated and capable of antimicrobial and toxic functions: damage to dopaminergic neurons can occur through reactive oxygen and nitrogen species, produced, respectively, by NADPH oxidase and inducible NO synthase (iNOS) [90]. As described above, activation of oxidative and nitrosative processes has been described in OSA. OSA, therefore, could be an additional insult on an already vulnerable brain, promoting the inflammatory neurodegenerative mechanisms and accelerating functional decline.

While no human studies exist looking at the locus coeruleus in OSA, animal data suggest OSA may reduce the noradrenergic locus coeruleus neuronal population and impair its function [70, 79, 117] (compare with sections on Intermittent hypoxemia and Sleep Fragmentation). While the focus of this review is on cognitive function, it can be inferred from the above that OSA, through its effects on the locus coeruleus, could affect the pathogenesis of PD. The implication is that OSA may not only promote decline in cognitive function, but also accelerate the overall disease process. This could include worsening of motor dysfunction in those with established PD and promoting development of overt PD in those with subclinical disease or with another predisposing factor (e.g., genetic). Indeed, recent epidemiological evidence suggests that OSA increases the risk of PD [118, 119].

## 7. Sleep and Cognitive Function in PD

Cognitive dysfunction is found in 20–40% of patients with early PD but is a major cause of long-term disability [113]. In one large study, after 20 years' follow-up, 83% of survivors had dementia [120]. The most commonly documented deficits in early PD are in executive “frontal” functions [121, 122] and memory [123, 124].

Sleep is a state that is crucial for proper cognitive function. It allows for consolidation of declarative memory [125] and of “implicitly” learned motor skills [126]. Implicit learning is dependent on attention [127] and is sensitive to sleep effects [128]. Poor sleep quality affects memory consolidation [129] and executive function [130, 131] in older adults. Changes in sleep EEG characteristics (sleep spindles and slow waves) with aging have been implicated in reduction in sleep-dependent memory consolidation in older adults [129].

Studies looking at sleep and sleep disorders in PD have found that subjective daytime sleepiness and fatigue are linked with cognitive impairment [132]. Presence of RBD is also linked with worse cognitive function [133, 134]. Poor sleep efficiency as measured by actigraphy has been variably associated with executive dysfunction [135] and memory deficits [136]. A recent meta-analysis has found multiple cognitive domains to be affected by poor sleep in PD [137], though most studies relied on self-reported sleep quality. Regarding implicit learning, PD patients appear not to have the expected improvement in motor skill following sleep [138, 139]. Hence, disrupted sleep, though a nonspecific symptom, appears to be an important factor in poor cognitive function and learning in PD. A recent study has found that specific sleep EEG (sleep spindle) alterations in PD are associated with subsequent development of dementia [140]. These alterations may be a marker of future dementia but it is unclear if sleep changes could be a causative factor in cognitive decline. Further work will need to be done to assess whether strategies aimed at improving sleep quality can reduce the risk of dementia.

## 8. Neuroimaging in relation to Cognitive Function in OSA and PD

Structural and functional changes on brain imaging associated with neurocognitive deficits have been found in OSA patients [23, 33, 141–143]. They include decreased grey matter in the hippocampus and temporal lobe, anterior cingulate, and cerebellum, as well as in the frontal and parietal lobes. CPAP therapy appears to increase gray-matter volume in hippocampal and frontal structures [33]. In PD, cortical atrophy in the hippocampus and frontal areas has been found in patients with mild cognitive impairment (MCI), but not in cognitively intact PD patients [144]. Most studies report a correlation of temporal lobe atrophy with poor memory in PD [145, 146], but some find a correlation between memory problems and frontal regions [147], or with medial temporal and frontal lobes [145]. It is conceivable that the variability in results is related at least partly to confounding effects of OSA, which was not accounted for in those studies. In that similar brain regions have been found to be affected in OSA, particularly temporal and frontal areas [33, 141]; OSA may contribute significantly to the cortical atrophy patterns identified in PD-MCI.

Functional neuroimaging in OSA has revealed decreased brain activation in cingulate, frontal, and parietal regions during performance of sustained attention and memory tasks [23, 141, 148]. In PD, poor performance on memory and executive function tests was associated with metabolic reductions in frontal and parietal association areas and relative increases in the cerebellar vermis and dentate nuclei, using FDG PET [149]. Other studies also report recruitment of additional pathways for the performance of certain cognitive tasks in PD, suggesting an adaptive compensatory response [150, 151], which has also been found in OSA [152, 153]. CPAP therapy, in one study [153], decreased OSA-related overactivation of prefrontal and hippocampal structures. Hence, both OSA and PD are independently associated with altered CNS activation during cognitive tasks, which may be reversible in the context of OSA. Activation patterns in patients with PD and OSA have not been studied.

## 9. Preliminary Data on Impact of OSA in PD

Little literature exists on outcomes related to OSA in PD. In one study, OSA was found to have a greater influence on memory consolidation in subjects with PD than in otherwise healthy OSA controls [154]. In another, working memory improvements after sleep showed a negative correlation with hypoxemia [155]. Our own preliminary data suggest that OSA is associated in PD patients with self-reported hypersomnolence and lower Montreal Cognitive Assessment (MoCA) scores [53], after adjusting for possible confounders. In an observational study, we have found that CPAP treatment of OSA led to an improvement in MoCA scores in PD patients with OSA but not those untreated or without OSA [156]. Neikrug et al., in the only RCT of OSA treatment in PD published to date, found that CPAP therapy was well tolerated and resulted in improved sleep architecture, as well as in reduced daytime sleepiness [157]. Despite the potential difficulties in applying CPAP therapy to PD patients, these promising results support further studies in this area.

## 10. Conclusion

Clearly, many questions remain and further work in this area will be necessary to clarify the role of OSA in PD. In a possible bidirectional relationship, OSA is potentially both a manifestation of PD, as well as a factor contributing to its signs and progression. Large prospective cohort studies will be needed to evaluate the impact of OSA on progression of PD-related cognitive dysfunction, as well as motor dysfunction. OSA has the merit of being largely correctable, such that effective treatment can readily improve its symptoms. RCTs will be needed to assess the effect of OSA therapies in PD. Treatment typically includes CPAP, though possibly other modalities could be more effective in PD than in the general population, given the somewhat different pathophysiology of OSA in PD. Moreover, if a deleterious effect of OSA on PD progression is confirmed, OSA treatment could be evaluated as a disease-modifying therapy, which could potentially delay cognitive decline or motor dysfunction.
